# Liquefied petroleum gas or biomass cooking and severe infant pneumonia

**DOI:** 10.1056/NEJMoa2305681

**Published:** 2024-01-04

**Authors:** Eric D. McCollum, John P. McCracken, Miles A. Kirby, Laura M. Grajeda, Shakir Hossen, Lawrence H. Moulton, Suzanne Simkovich, Dina Goodman-Palmer, Ghislaine Rosa, Alexie Mukeshimana, Kalpana Balakrishnan, Gurusamy Thangavel, Sarada S. Garg, Adly Castañaza, Lisa M. Thompson, Anaité Diaz-Artiga, Aris T. Papageorghiou, Victor G. Davila-Roman, Lindsay Underhill, Stella Hartinger, Kendra N. Williams, Laura Nicolaou, Howard H. Chang, Amy E. Lovvorn, Joshua Rosenthal, Ajay Pillarisetti, Wenlu Ye, Luke P. Naeher, Michael A. Johnson, Lance A. Waller, Shirin Jabbarzadeh, Jiantong Wang, Yunyun Chen, Kyle Steenland, Thomas F. Clasen, Jennifer L. Peel, William Checkley

**Affiliations:** 1. Global Program in Pediatric Respiratory Sciences, Eudowood Division of Pediatric Respiratory Sciences, School of Medicine, Johns Hopkins University, Baltimore, MD, USA; 2. Department of International Health, Bloomberg School of Public Health, Johns Hopkins University, Baltimore, MD, USA; 3. Global Health Institute, Epidemiology and Biostatistics Department, University of Georgia, Athens, GA, USA; 4. Center for Health Studies, Universidad del Valle de Guatemala, Guatemala City, Guatemala; 5. Department of Global Health and Population, Harvard T.H. Chan School of Public Health, Boston, MA, USA; 6. Division of Pulmonary and Critical Care, School of Medicine, Johns Hopkins University, Baltimore, MD, USA; 7. Center for Global Non-Communicable Disease Research and Training, School of Medicine, Johns Hopkins University, Baltimore, MD, USA; 8. Program in Global Disease Epidemiology and Control, Department of International Health, Bloomberg School of Public Health, Johns Hopkins University, Baltimore, MD, USA (Now with Pfizer Canada ULC); 9. Division of Healthcare Delivery Research, MedStar Health Research Institute, Hyattsville, USA; 10. Division of Pulmonary and Critical Care Medicine, Georgetown University, Washington D.C., USA; 11. University of Liverpool, Liverpool, UK; 12. Eagle Research Center Limited, Kigali, Rwanda; 13. ICMR Center for Advanced Research on Air Quality, Climate and Health, Department of Environmental Health Engineering, Sri Ramachandra Institute for Higher Education and Research, Chennai, India; 14. Nell Hodgson Woodruff School of Nursing, Emory University, Atlanta, GA, USA; 15. Nuffield Department of Women’s & Reproductive Health, University of Oxford, Oxford, UK; 16. Global Health Center, Institute for Public Health and Cardiovascular Division, Department of Medicine, Washington University in St. Louis, MO, USA; 17. Department of Biostatistics and Bioinformatics, Rollins School of Public Health, Emory University, Atlanta, GA, USA; 18. Gangarosa Department of Environmental Health, Rollins School of Public Health, Emory University, Atlanta, GA, USA; 19. Fogarty International Center, National Institutes of Health, Bethesda, MD, USA; 20. Division of Environmental Health Sciences, School of Public Health, University of California at Berkeley, Berkeley, CA, USA; 21. Department of Environmental Health Science, College of Public Health, University of Georgia, Athens, Georgia, USA; 22. Berkeley Air Monitoring Group, Berkeley, CA, USA; 23. Department of Environmental and Radiological Health Sciences, Colorado State University, Fort Collins, CO, USA

## Abstract

**Background:**

Household air pollution exposure is a risk factor for severe pneumonia. The effect of replacing biomass cooking with liquefied petroleum gas (LPG) cookstoves on severe infant pneumonia is uncertain.

**Methods:**

We conducted a randomized controlled trial among 3,200 pregnant women aged 18-34 years and 9 to <20 weeks gestation in India, Guatemala, Peru, and Rwanda May 2018–September 2021. Pregnant women were randomized to unvented LPG stoves and fuel (intervention) or continued biomass fuel cooking (control). We monitored intervention adherence and measured 24-hour personal exposure to fine particulate matter (PM_2.5_) in pregnant women and their offspring. The trial had 4 primary outcomes; the primary outcome described in the present report was severe pneumonia in the first year of life, identified by facility surveillance or verbal autopsy of deaths.

**Results:**

We randomized 3,195 pregnant women who gave birth to 3,061 infants. High intervention uptake led to reduced PM_2.5_ personal exposures among children (intervention median 24.2 μg/m^3^ (interquartile range (IQR) 17.8, 36.4); control median 66.0 μg/m^3^ (IQR 35.2, 132.0). There were 175 severe pneumonia episodes identified during the first year of life, with an incidence rate of 5.67 (95% confidence interval (CI) 4.55, 7.07) and 6.06 (4.81, 7.62) cases per 100-child years in intervention and controls (incidence rate ratio 0.96 [98.75% CI, 0.64, 1.44; p=0.81]. No severe adverse events associated with the intervention were reported.

**Conclusions:**

There was no significant difference in severe pneumonia incidence among infants of women randomized to LPG compared to biomass-burning cookstoves.

## Introduction

Pneumonia is a leading cause of child mortality worldwide, with most deaths in infants^1^. About 83% of the 808,000 annual child pneumonia deaths occur in sub-Saharan Africa, South Asia, and Latin America^1^. Observational studies suggest fine particulate air pollution (PM_2.5_) exposure from incomplete solid fuel combustion is a risk factor for pneumonia^1^. Nearly ~30% of global pediatric pneumonia deaths are attributed to household air pollution^1^. About 2.4 billion people – predominantly in low- and middle-income countries (LMICs) – use biomass (wood, charcoal, animal dung, coal) daily to cook or heat their households^2^.

To date, randomized controlled trials (RCTs) of cleaner cooking interventions have not found an effect on primary child pneumonia outcomes^3–6^. However, it is unclear if lack of benefit stemmed from insufficiently lowered pollutant levels due to inadequate cookstove intervention uptake or performance, lack of specificity in pneumonia case definitions, or low statistical power. The Household Air Pollution Intervention Network (HAPIN) trial was designed to address these limitations in assessing whether cooking with an unvented liquefied petroleum gas (LPG) stove and fuel during pregnancy and the offspring’s first year of life, compared to biomass, reduced severe infant pneumonia incidence and other health outcomes^7^. We previously reported no evidence of an intervention effect on birthweight^8^. Here, we report severe pneumonia incidence during the first year of life, one of four primary trial outcomes.

## Methods

### Design

HAPIN was a randomized controlled trial of unvented LPG cookstoves with free, uninterrupted fuel supply, compared to usual cooking practices (primarily or exclusively with biomass fuels), conducted in Tamil Nadu, India; Jalapa, Guatemala; Puno, Peru; and Kayonza, Rwanda from May 2018–September 2021^7^. Sites were selected to cover a range of geographical settings in four continents where biomass is used for cooking.

### Participants

Pregnant women aged 18–34 years with an ultrasound and pregnancy-test confirmed, viable, singleton fetus at 9 to <20 weeks of gestation, biomass stove use at least 4 days a week, and study area residency, were eligible. Pregnant women who smoked tobacco, planned to migrate from the study area during the study, or used or planned to switch to LPG stoves were excluded. One pregnant woman per household could be enrolled.

### Randomization

We randomized participants to intervention and control groups on a 1:1 basis. India and Peru used stratified randomization to ensure balance between two and six distinct geographical study areas, respectively. While the intervention assignment could not be blinded to participants and field staff, all investigators were masked to study group at the time of data cleaning, image interpretation, or data analysis.

### Intervention

Unvented LPG cookstoves all had ≥2 burners and met local safety standards. Behavioral reinforcement messaging was provided to foster exclusive, safe LPG stove use^9^. Staff monitored both groups for adherence to group allocation through stove temperature sensor monitoring^10^. Controls were provided non-monetary compensation to counterbalance the intervention incentive of free fuel provision and mitigate attrition^11^. As cooking fuel delivery was considered an essential service, the intervention was generally uninterrupted by COVID-19 restrictions with similar delivery times before and during COVID^12^.

### Exposure assessment

Twenty-four-hour personal exposure to PM_2.5_, carbon monoxide, and black carbon were measured directly using wearable devices for pregnant women at baseline (<20 weeks) and 24-28 and 32-36 weeks’ gestation^10^. We estimated infants’ exposure to PM_2.5_, carbon monoxide, and black carbon at 1-3, 6 and 12 months of age using an indirect method^13^ (Supplementary Appendix).

### Outcome Surveillance

We conducted active surveillance of severe pneumonia cases at pre-selected community hospitals and health centers. These facilities were identified during formative work as centers where severe cases receive care^14^. Passive facility and household surveillance were also conducted to identify missed facility visits, missed hospitalizations, ventilatory support, and deaths. Study staff were trained to evaluate children for severe pneumonia using a standard approach^15^; in brief, they passed certification examinations and received annual re-trainings. If medical care was needed, mothers could notify HAPIN staff by phone to facilitate appropriate care. In India, Peru, and Rwanda, study staff were available weekdays in-person at sentinel facilities and by phone anytime; in Guatemala, staff were available in-person continuously at the sentinel hospital. We reviewed medical charts of infant deaths and conducted a verbal autopsy to determine whether the death was related to severe pneumonia. Beginning in November 2019, sites in Rwanda increased study staff presence at outpatient clinics as surveillance identified some cases who were not hospitalized. In March 2020, COVID-19-related public health measures commenced at all sites, which limited active in person surveillance and care-seeking during lockdown periods. HAPIN staff also telephoned facility contacts to surveil for possible cases; telephone surveillance was uninterrupted during the study.

### Outcomes

The primary outcome was severe pneumonia incidence in the first year of life among participant offspring. The primary case definition was adapted from World Health Organization (WHO) guidelines based on external expert input.^16^. In July 2019, when follow-up time of infants was <1%, we implemented additional expert recommendations to amend the case definition and improve specificity, objectivity, and to be responsive to formative data we collected (Supplementary Appendix)^17,18^ The primary definition of severe pneumonia was defined as (1) cough and/or difficult breathing, ≥1 general danger sign (unable to drink or breastfeed, vomiting everything, convulsions, stridor at rest, lethargy or unconscious) or ≥1 neonatal danger sign (unable to feed well, not moving at all or movement only when stimulated, grunting, severe chest indrawing), and pneumonia on imaging, (2) cough and/or difficult breathing with hypoxemia, or (3) a verbal autopsy-confirmed pneumonia death.^15^ Subsequent symptoms in the same child were considered separate episodes if >14 days after hospital discharge or >30 days from outpatient diagnosis. To be eligible as a case required examination by study staff except for children on ventilatory support or who died.

Chest imaging was by ultrasound (Sonosite Edge, Bothell, WA, USA)^15,19^ or radiography if ultrasound was unavailable. The reported sensitivity and specificity of lung ultrasound for diagnosing pneumonia in children are 95.5% and 95.3%, respectively and for chest radiography are 86.8% and 98.2%, respectively. ^20^. All images were interpreted by adjudication panels blinded to intervention and clinical status^15,19,21^. Two panelists followed pre-specified interpretation procedures and were required to agree on the presence or absence of pneumonia for the image to be classified as pneumonia. Pneumonia on imaging was a consolidation alone (meeting pre-specified size dimensions), or a pleural effusion near an infiltrate, or pleural abnormalities (ultrasound-specific)^15,19,21^.

Hypoxemia was defined as a peripheral arterial oxyhemoglobin saturation (SpO_2_) ≤92% at <2,500 meters altitude (Guatemala, India, Rwanda) or ≤86% at ≥2,500 meters altitude (Peru)^15^, or receipt of invasive or non-invasive ventilation or high-flow oxygen. To measure SpO_2_, facility study staff applied a Masimo Rad-G^®^ pulse oximeter (Masimo, Irvine, CA, USA) and pediatric probe to the big toe of infants breathing room air. Staff collected three measurements over two-minutes, and these were averaged. SpO_2_ measurements were extracted from medical charts when available.

Trained, local medical staff performed verbal autopsies with caregivers of deceased infants using a validated protocol^22^. A physician verbal autopsy panel assigned primary and secondary causes of death using WHO 2016 ICD-10 codes. Two non-study LMIC physicians masked to randomization and other death classifications independently reviewed the autopsy open narrative and closed questions. When the assigned primary cause of death was discordant between the two physicians, a pediatrician panelist arbitrated to achieve consensus. Cases without consensus were undetermined. The final verbal autopsy classification was pneumonia if it was the primary or secondary cause of death.

Secondary outcomes were pneumonia per WHO Integrated Management of Childhood Illness (IMCI) guidelines and WHO Pocketbook guidelines^23,24^, hypoxemia and/or imaging-confirmed pneumonia, and any hospitalized respiratory illness (see Table S1 for secondary outcome definitions).

### Statistical analysis

Based on available evidence^5,6,25–28^, we estimated a sample of 3,200 pregnant women would provide 80% power to detect a 36% reduction in severe pneumonia incidence between study arms assuming a baseline rate of 9/100 infant-years using an α of 0.0125 (multiple hypothesis testing for four trial outcomes)^7^. The primary analysis was according to intention-to-treat and was conducted independently by two teams. We used Poisson regressions with generalized estimating equations (GEE) to model the incidence of all episodes of severe pneumonia using infant days at risk as the denominator to derive incidence rate ratios (IRRs). The intervention arm was the main covariate and models were adjusted for 10 randomization strata (one in Guatemala and Rwanda, two in India, six in Peru). The threshold for statistical significance for the primary outcome was set *a priori* at 0.0125 to account for the four primary trial outcomes. When data were incomplete for an outcome classification, we assumed the event did not occur.

Secondary analyses estimated the intervention effect on the time to first pneumonia incidence via Cox proportional hazards models. Subgroup analyses assessed the influence of outpatient surveillance changes in Rwanda (after November 2019) and the COVID-19 pandemic (after March 2020) using GEE Poisson regression models with indicator variables for relevant time periods, as well as interaction terms between treatment arms and time periods to assess whether the intervention effect changed over time. Given the clustering of deaths very early in life, and that diagnostic accuracy may be lower in neonates, we also conducted sensitivity analyses of the primary analysis that excluded cases <7 and <30 days old.

### Oversight

The protocol, available at NEJM.org, was approved by all investigator-affiliated institutional review boards (see Appendix). Participants provided written informed consent. An independent data and safety monitoring board (DSMB) monitored safety and efficacy and received unblinded interim analyses. No pre-defined stopping rules were formulated due to the low intervention risk. Eric D. McCollum, William Checkley, John McCracken, Jennifer Peel, and Thomas Clasen take responsibility for the integrity and completeness of the data and fidelity of the report to the protocol.

## Results

### Participant characteristics

**Figure 1.**
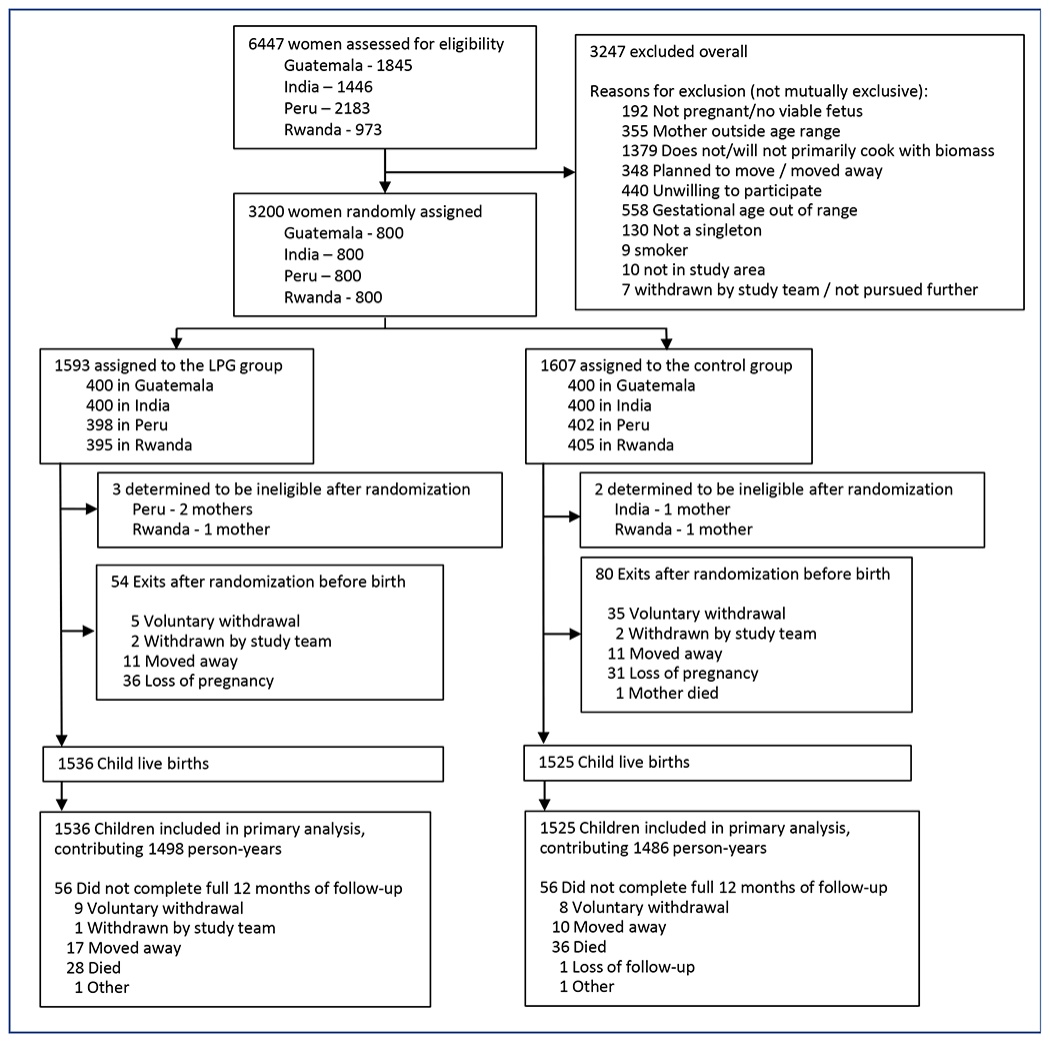
CONSORT diagram.

3,200 women were randomized, with 1,593 (49.8%) allocated to the LPG arm and 1,607 (50.2%) as controls (Figure 1). Baseline maternal characteristics were similar between groups (Table 1); the pregnant women and their offspring were representative of the broader population of women and infants affected by indoor air pollution from biomass cooking (Table S2). Pregnant women received LPG stoves mid-second trimester (mean 18.1 weeks (SD, 3.3). There were 1,536 livebirths overall in the intervention group and 1,525 in controls. Table 2 reports the characteristics of liveborn children by study group, including vaccination status.

### Intervention fidelity, adherence, and effects on exposure

Intervention participants used biomass stoves on a median of 0.4% (interquartile range (IQR) 0.0, 2.3) of monitored days^12,29^. The averaged, post-randomization, 24-hour personal exposures to PM_2.5_ were overall lower in the intervention arm, compared to controls in the antenatal (median 24.8 μg/m^3^, interquartile range (IQR) 17.0, 40.5 vs median 77.0 μg/m^3^, 40.7, 132.8) as well as during postnatal periods (median 24.2 μg/m^3^, IQR 17.8, 36.4 vs median 66.0 μg/m^3^, IQR 35.2, 132.0) (Table 2, Table S3)^13,30^.

### Primary outcome

**Figure 2.**
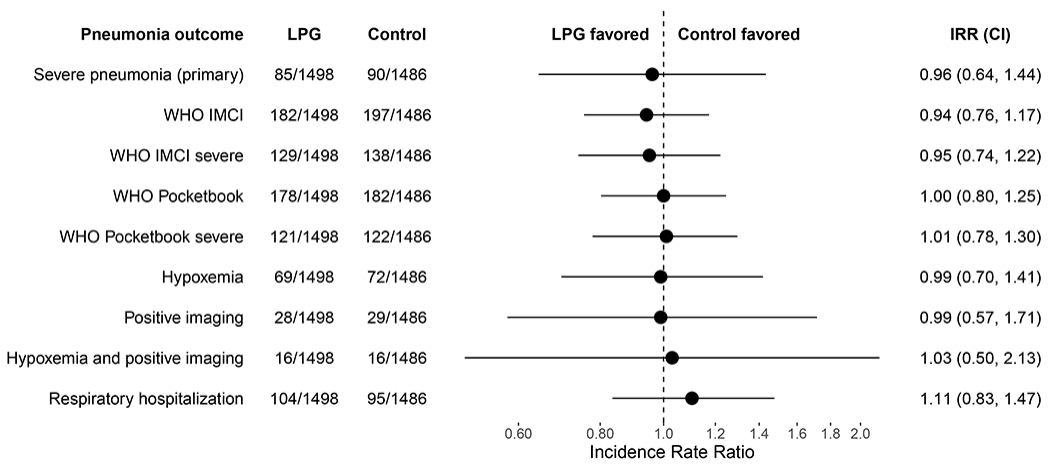
Intervention effects on primary and secondary outcomes. LPG indicates liquefied petroleum gas; IRR, Incidence Rate Ratio; IMCI, Integrated Management of Childhood Illnesses; WHO, World Health Organization. See Appendix Table S1 for secondary outcome case definitions. Severe pneumonia (primary) 98.75% confidence interval was adjusted for multiplicity. 95% confidence intervals of other endpoints were not adjusted for multiplicity and should not be used to infer definitive treatment effects.

We identified 85 severe pneumonia episodes in the intervention group and 90 in the control group (Figure 2) from 1,243 healthcare facility visits and 55 verbal autopsies (Figure S1, Tables S4–S8). Among these, there were 12 deaths attributed to pneumonia (6.9% of pneumonia outcomes), eight in the control group and 4 in the intervention group.) (Table S9). The severe pneumonia incidence rate in the first year of life was 5.67 (95% CI 4.45, 7.07) per 100 child-years in the LPG group and 6.06 (95% CI 4.81, 7.62) per 100 child-years in the control group (incidence rate ratio (98.7% CI) 0.96 (0.64, 1.44; p=0.81) (Figure 2).

### Secondary outcomes, subgroup and sensitivity analyses

**Figure 3.**
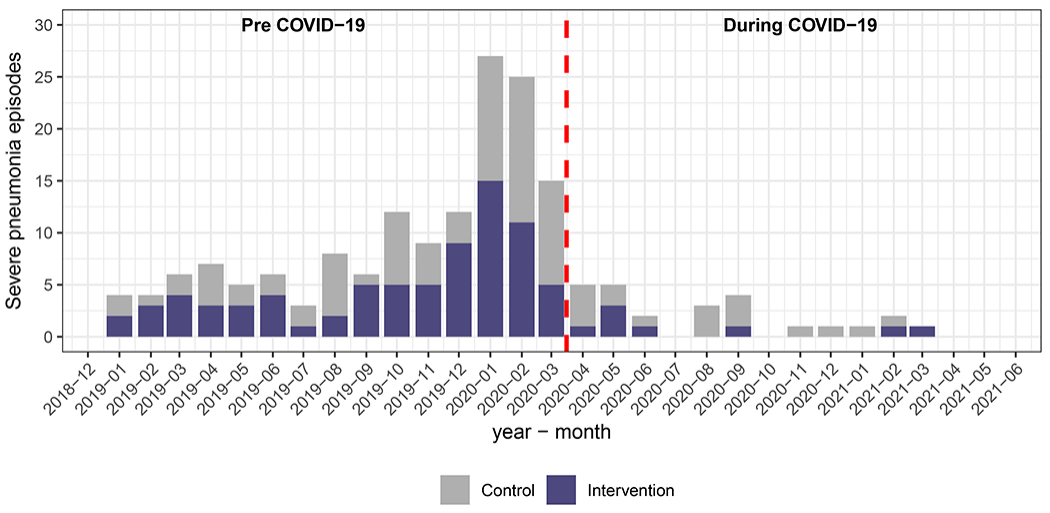
Episodes and incidence of severe pneumonia over time.

No evidence of an intervention effect was observed for secondary outcomes (Figure 2, Table S10) or when stratified by study site or other subgroups (Figure S2). Although the observed incidence of severe pneumonia across all study sites decreased by 77% (95% CI 61%, 86%) during the COVID-19 pandemic period (Figure 3, Figure S3, Table S11), there was no appreciable change in the IRR when our models accounted for the pandemic period and child’s age (IRR 0.96, 95% CI 0.70, 1.31). The IRRs before (0.71 IRR, 95% CI 0.12, 4.23) and after (IRR 0.80, 95% CI 0.49, 1.31) surveillance changes (November 2019) in Rwanda were also similar (Table S12).

### Adverse Events

Burns were reported by three infants (0.2%) in the intervention group and seven infants (0.5%) in the control arm. No burn was classified as a serious adverse event Table S13).

## Discussion

Despite high LPG intervention uptake and substantial reductions in air pollutant exposure, we found no significant difference in the incidence of severe infant pneumonia between the intervention and control arms in this multi-country trial. Our findings are consistent with null findings from a cluster randomized trial in Ghana of a similar cookstove^3^, indicating that unvented LPG cookstoves are unlikely to reduce severe infant pneumonia. Our trial also found no difference between study arms in the other primary endpoints of birthweight^8^ and stunting (reported in another article in this issue of the *Journal*,) ^31^.

There are several potential explanations for our null findings for severe infant pneumonia. First, evidence suggests household air pollution is more closely linked with bacterial than viral nasopharyngeal carriage^32,33^. While nasopharyngeal carriage is considered a prerequisite for the development of invasive or mucosal bacterial diseases like pneumonia^34^, populations vaccinated against Haemophilus Influenzae type B (Hib) and Streptococcus pneumoniae (pneumococcus) are well protected from nasopharyngeal carriage progressing to disease^35^. Our study population had high rates of vaccination against Hib and pneumococcal pneumonia, making severe bacterial pneumonia less likely. As observed in this trial (Figure 3, Figure S3) and elsewhere, the fact that mitigation efforts during the COVID-19 pandemic dramatically reduced both respiratory virus circulation and pediatric hospitalizations provides indirect evidence on the central role of viruses in severe childhood respiratory disease^36,37^. However, definitively determining the etiology of severe childhood pneumonia is challenging, and we do not have information on respiratory pathogens in these infants.

Second, the PM_2.5_ levels we achieved were lower than levels in other trials^3–6^ but remained above WHO recommendations^38^. Although uncertain, it is possible that lower PM_2.5_ exposure levels than were achieved may be required to reduce the risk of severe pneumonia and greater reductions may require broader community interventions, rather than household strategies as we employed. Third, even though unvented LPG cookstoves produce nitrogen dioxide (NO_2_) at levels lower than biomass cookstoves, these levels are nevertheless above recommendations^39^. Elevated NO_2_ concentrations have associations with asthma in children^40^, and may have contributed to our null results.

Limitations of our study should be noted. Incomplete assessments at facility visits may have led to missed cases, although this is unlikely to have impacted our results because missingness among screened children was low (Figure S1). It is also possible that incomplete case ascertainment occurred due to children seeking care at clinics outside of the surveillance area or failing to seek care at all. Missed cases may have been more common during the COVID-19 pandemic period, particularly in the first months during lockdowns. We accounted for the pandemic in our analysis but did not find evidence of differential effects of the pandemic on our results (Table S11). The wide confidence intervals around our effect estimates mean that we cannot exclude clinically important reductions or increases in severe pneumonia risk with the use of unvented LPG cookstoves compared to biomass cookstoves. Also since there is no gold standard for pneumonia diagnosis, the accuracy of our primary case definition for severe pneumonia is undetermined. However, we sought and incorporated external expert recommendations intended to optimize the definition’s objectivity and specificity. The results for outcomes using alternative pneumonia definitions were also consistent with results for the primary outcome.

In conclusion, in this multicenter trial involving four LMICs, unvented LPG cookstoves did not reduce the incidence of severe infant pneumonia compared to biomass cookstoves.

## Supplementary Material

1

## Figures and Tables

**Table 1.** Baseline characteristics of pregnant women (with liveborn children) by study group

Characteristic		Intervention (N=1536)	Control (N=1525)
IRC, n (%)	Guatemala	384 (25.0%)	386 (25.3%)
	India	388 (25.3%)	387 (25.4%)
	Peru	385 (25.1%)	358 (23.5%)
	Rwanda	379 (24.7%)	394 (25.8%)
	Missing, n	0	0
Mother’s age (years) at baseline	Mean (SD)	25.3 (4.4)	25.4 (4.5)
	18 to <25 years, n (%)	787 (51.2%)	758 (49.7%)
	25 to <30 years, n (%)	484 (31.5%)	488 (32.0%)
	30 to <35 years, n (%)	265 (17.3%)	279 (18.3%)
	Missing, n	0	0
Mother’s highest level of education completed, n (%)	None or some primary	461 (30.0%)	540 (35.4%)
	Primary or some secondary	538 (35.0%)	514 (33.7%)
	Secondary, vocational, or university/college	537 (35.0%)	471 (30.9%)
	Missing, n	0	0
Gestational age (weeks) at baseline	Mean (SD)	15.5 (3.1)	15.3 (3.2)
	Missing, n	0	0
Gestational age (weeks) at intervention^1^	Mean (SD)	18.1 (3.3)	17.9 (3.2)
	10 to <18 weeks	767 (49.9%)	791 (51.9%)
	18 to <30 weeks	769 (50.1%)	734 (48.1%)
	Missing, n	0	0
Number of siblings in the household	Mean (SD)	1.0 (1.1)	1.1 (1.2)
	Missing, n	0	0
Second-hand Smoking	n (%)	146 (9.5%)	174 (11.4%)
	Missing, n	1	2
Household food insecurity score^2^, n (%)	Food secure	904 (58.9%)	820 (53.8%)
	Mild	403 (26.2%)	424 (27.8%)
	Moderate/severe	208 (13.5%)	259 (17.0%)
	Missing, n	21	22
Socioeconomic status index^3^	Mean (SD) (range)	−0.1 (1.1) (−2.2, 2.1)	0.1 (1.0) (−2.2, 2.1)
	Missing, n	0	0
PM_2.5_ (μg/m^3^)^4^	Median (IQR)	81.6 (45.9, 150.7)	84.2 (46.5, 143.0)
	Missing, n	184	173
Black carbon (μg/m^3^)^4^	Median (IQR)	10.5 (6.2, 15.4)	10.9 (6.9, 15.5)
	Missing, n	313	314
Carbon monoxide (ppm)^4^	Median (IQR)	1.3 (0.5, 3.0)	1.2 (0.5, 2.5)
	Missing, n	152	150

IRC indicates International Research Center; SD, standard deviation; PM, particulate matter; IQR, interquartile range.

1 Control group calculated as gestational age at baseline plus 2.6 weeks, which is the average time between baseline and stove installation in the intervention group.

2 Categories (corresponding score): Food secure (0); Mild (1,2,3), Moderate (4,5,6) or Severe (7,8) food insecurity.

3 Principal component analysis was applied to data on number of people in the household, participant’s education level, quality of water and sanitation, access to electricity, housing materials, ownership of 24 specific household assets and food insecurity at the start of the study. Multiple imputation with chained equations was used to handle missing data. A higher index indicates worse socioeconomic status.

4 Missing includes invalid samples that failed to pass quantitative quality checks, including samples with unacceptable flow rates, filter damage, and measurement durations outside of 24 ± 4 hours.

**Table 2.** Characteristics of liveborn children by study group

Characteristic		Intervention (N=1536)	Control (N=1525)
Child sex, n (%)	Male	800 (52.1%)	787 (51.6%)
	Female	736 (47.9%)	738 (48.4%)
	Missing, n	0	0
Birth weight-for-age z score	Mean (SD)	−0.8 (1.0)	−0.8 (1.0)
	Missing, n	24	3
Exclusive breastfeeding^1^	n (%)	702 (48.9%)	747 (52.5%)
	Missing, n	100	101
Up-to-date pentavalent vaccine at study exit^2^	n (%)	1306 (95.4%)	1311 (95.2%)
	Missing, n	167	148
Up-to-date pneumococcal conjugate vaccine at study exit^2^	n (%)	999 (97.6%)	1000 (96.8%)
	Missing, n	513	492
Trial-period antenatal PM_2.5_ (μg/m^3^)^3^	Median (IQR)	24.8 (17.0, 40.5)	77.0 (40.7, 132.8)
	Missing, n	99	116
Trial-period postnatal PM_2.5_ (μg/m^3^)^3^	Median (IQR)	24.2 (17.8, 36.4)	66.0 (35.2, 132.0)
	Missing, n	688	592
Trial-period antenatal black carbon (μg/m^3^)^3^	Median (IQR)	2.9 (1.7, 4.8)	10.0 (5.9, 14.1)
	Missing, n	123	149
Trial-period antenatal carbon monoxide (ppm)^3^	Median (IQR)	0.3 (0.1, 0.8)	1.2 (0.5, 2.4)
	Missing, n	86	95
Trial-period postnatal carbon monoxide (ppm)^3^	Median (IQR)	0.3 (0.0, 0.8)	1.3 (0.4, 3.0)
	Missing, n	571	609

IRC indicates International Research Center; SD, standard deviation; PM, particulate matter; IQR, interquartile range.

1 During the first six months of life feeding only breast milk, not any other foods or liquids including infant formula or water.

2 Up-to-date vaccination at one year old when received three doses of the pentavalent vaccine for all IRCs, three doses of PCV for Rwanda, or two doses of PCV for Guatemala and Peru. PCV was not available in India.

3 Trial-period measurements refer to post-randomization pollutant values, which are presented as the median of the average of household-level measures. Missing includes invalid samples that failed to pass quantitative quality checks, including samples with unacceptable flow rates, filter damage, and measurement durations outside of 24 ± 4 hours. Post-natal black carbon measurement is not available.
