# Supplementary material for: Liquefied petroleum gas or biomass cooking and severe infant pneumonia
Source: N Engl J Med. Author manuscript; Available in PMC 2024 Jan 5. (PMC10768798; doi:10.1056/NEJMoa2305681)
Supplement: 1 [file NIHMS1945621-supplement-1.pdf]

Supplementary Appendix

Liquefied petroleum gas or biomass cooking and severe infant pneumonia

Table of contents:

I. **HAPIN Investigators** – page 2

II. **Methodology**
- **Ethical review approvals** – page 2
- **Indirect exposure assessment for children** – page 4
- **HAPIN Pneumonia Meeting Minutes: case definition amendment** – page 6
- **Verbal autopsy methodology** – page 11

III. **Tables**
- **Table S1. Secondary outcome definitions** – page 16
- **Table S2**. **Background information on the broader population affected by household air pollution** – page 18
- **Table S3. Air pollution exposure measurements** – page 19
- **Table S4. Verbal Autopsy – Summary of physician coding process** – page 21.
- **Table S5. Verbal Autopsy – Primary cause of death, assigned from different methods** – page 22
- **Table S6. Verbal Autopsy – Probable pneumonia deaths, assigned through different verbal autopsy analysis methods** – page 23
- **Table S7. Verbal Autopsy – Output data dictionary** – page 24
- **Table S8. Verbal Autopsy – ICD-10 derived cause of death codes** – page 26
- **Table S9. Characteristics of severe pneumonia cases (primary outcome) by study group** – page 27
- **Table S10**. **Time to event analysis – secondary analysis** – page 31
- **Table S11**. **Primary pneumonia analysis accounting for COVID-19 period and child´s age – secondary analysis** – page 32
- **Table S12. Rwanda – secondary analysis** – page 33
- **Table S13. Adverse events** – page 34

IV. **Figures**
- **Figure S1. Primary pneumonia case ascertainment schema** – page 35
- **Figure S2. Subgroup analyses** – page 36
- **Figure S3. Episodes and incidence of severe pneumonia and health care visits by COVID-19 period** – page 37

V. **References – page 38**

Correspondence:

Eric D. McCollum, MD, MPH
Global Program in Pediatric Respiratory Sciences
Eudowood Division of Pediatric Respiratory Sciences
Johns Hopkins University
200 N. Wolfe St, Suite 3071

William Checkley, MD, PhD
Division of Pulmonary and Critical Care
Johns Hopkins University
1830 E. Monument St, Suite 555
Baltimore, MD 21287

Baltimore, MD 21287
Email: emccoll3@jhmi.edu
Email: wcheckl1@jhmi.edu

I. HAPIN Investigators

Vigneswari Aravindalochanan, Gloriose Bankundiye, Dana Boyd Barr, Vanessa Burrowes, Alejandra Bussalleu, Devan Campbell, Eduardo Canuz, Marilú Chiang, Maggie L. Clark, Rachel Craik, Mary Crocker, Lisa de las Fuentes, Oscar De León, Ephrem Dusabimana, Lisa Elon, Juan Gabriel Espinoza, Irma Sayury Pineda Fuentes, Ahana Ghosh, Savannah Gupton, Sarah Hamid, Steven A. Harvey, Mayari Hengstermann, Ian Hennessee, Phabiola Herrera, Marjorie Howard, Penelope P. Howards, Lindsay Jaacks, Katherine Kearns, Jacob Kremer, Margaret A. Laws, Pattie Lenzen, Jiawen Liao, Jane Mbabazi, Julia N. McPeek, Rachel Meyers, J. Jaime Miranda, Erick Mollinedo, Libny Monroy, Krishnendu Mukhopadhyay, Bernard Mutariyani, Luke Naeher, Abidan Nambajimana, Durairaj Natesan, Florien Ndagijimana, Laura Nicolaou, Azhar Nizam, Jean de Dieu Ntivuguruzwa, Ricardo Piedrahita, Naveen Puttaswamy, Elisa Puzzolo, Ashlinn Quinn, Karthikeyan Dharmapuri Rajamani, Sarah Rajkumar, Usha Ramakrishnan, Rengaraj Ramasami, Alexander Ramirez, P. Barry Ryan, Sudhakar Saidam, Zoe Sakas, Sankar Sambandam, Jeremy A. Sarnat, Sheela S. Sinharoy, Kirk R. Smith, Damien Swearing, Ashley Toenjes, Jean Damascene Uwizeyimana, Viviane Valdes, Amit Verma, Megan Warnock, Wenlu Ye, Bonnie N. Young, Ashley Younger.

II. Methodology

Ethical review approvals

The trial protocol, available with the full text of this article at NEJM.org, was reviewed and approved by the institutional review boards or ethics committees of Emory University, Johns Hopkins University, the Sri Ramachandra Institute of Higher

Education and Research, Universidad del Valle de Guatemala, Asociación Benéfica PRISMA, the London School of Hygiene and Tropical Medicine, and Washington University in St. Louis and by the Indian Council of Medical Research – Health Ministry Screening Committee, the Guatemalan Ministry of Health National Ethics Committee, and the Rwandan National Ethics Committee.

Indirect exposure assessment for children

Due to the difficulty in instrumenting small children with particulate matter (PM) measuring equipment, we utilized a validated time-activity and microenvironmental concentration exposure reconstruction method to estimate children's exposure to fine PM ($PM_{2.5}$) and carbon monoxide (CO)[1]. The method relies on objective minute-by-minute measurement of location using Bluetooth beacons (see below) and minute-by-minute measurement of pollutant concentrations in commonly frequented environments. Exposures are reconstructed by estimating a time-weighted average based on the pollutant concentrations in each environment in which participants spend time[2]. We attempted collection of three 24-hour $PM_{2.5}$ and CO measurements for children in the first year of life (<3 months, ~6 months, and ~12 months).

Microenvironmental $PM_{2.5}$ and CO measurements

Microenvironmental $PM_{2.5}$ concentrations were measured with Enhanced Children's MicroPEMs (ECM, RTI International, Durham, NC USA). ECMs measure $PM_{2.5}$ concentrations every 10 seconds via light-scattering and collect integrated samples on 15 mm PTFE filters (Measurement Technology Laboratories)[2]. Gravimetrically-corrected nephelometric $PM_{2.5}$ concentrations are used in infants' indirect $PM_{2.5}$ exposure estimates. We used the Lascar EL-USB-300 (Lascar Electronics) to measure continuous CO concentrations at 1-minute intervals. The Lascar is a large pen-sized device with a sensing range between 0 and 300 ppm. Details on deployment and analyses of data from these instruments have been described previously[2,3]. Monitored microenvironments were the most commonly occupied rooms (i.e., the main

living/sleeping areas, the kitchen), and mothers who consented to be a mobile microenvironment and wore an exposure monitor and beacon logger[2].

Indirect exposure measurements

We used Bluetooth®-based beacons for estimating in which microenvironments infants spent time. The beacon system consists of Bluetooth emitters (Model O, Roximity Inc. Denver, CO, USA or EM Microelectronic, La Tène, Neuchâtel, Switzerland) and bluetooth signal loggers (Berkeley Air Monitoring Group, Berkeley, CA, USA). The emitter (hereafter 'beacon') is a coin-size device that constantly emits signals[4]; infants wore two Beacons sewed into their clothing. The Beacon logger is a smartphone-sized device that receives and logs Bluetooth signals emitted from the Beacons[4]. The logger records the beacon's unique media access control (MAC) address and the received signal strength indicator (RSSI) every 20 seconds. RSSI is proportional to the distance between a Beacon and a logger and is used to determine the infant's location[4].

In each microenvironment, we co-located a logger with $PM_{2.5}$ and CO monitors. At 5 minute intervals, we assigned location to the area with the strongest average RSSI from the two beacons worn by the infant[4]. Reconstructed personal $PM_{2.5}$/CO exposures for infants were estimated by integrating corresponding microenvironmental concentrations with time spent in the respective locations within the 24-hour monitoring period.

HAPIN Pneumonia Meeting Minutes: case definition amendment

Meeting: HAPIN Pneumonia Meeting Minutes
Dates: July 25 and 26, 2019
Location: Bill and Melinda Gates Foundation, Seattle, USA

Lead facilitator: Dr. Eric D. McCollum
Attendees: Dr. Thomas Clasen, Dr. Kyle Steenland, Dr. Will Checkley, Dr. Lisa Thompson, Dr. John McCracken, Dr. Ghislaine Rosa, Dr. Suzanne Simkovich, Ms. Shirin Jabbarzadeh,
External experts: Dr. Harry Campbell, Dr. Heather Zar, Dr. Claudio Lanata

Summary

Meeting objectives:

The main objectives of the meeting were to (1) provide an update on HAPIN pneumonia progress, (2) present pneumonia data from PINS (Pneumonia International Network Surveillance) and HAPIN (Household Air Pollution International Network), (3) re-evaluate pneumonia trial case definition and make recommendations with the input of external child pneumonia experts.

HAPIN trial pneumonia data:

The main HAPIN trial data was presented based on data through June 20, 2019. The intervention status of the cases remained concealed for this analysis, the meeting, and all investigators and meeting participants maintained blinding at all times. These data represented <1% of the total child-months follow-up for children <1 year old across all the International Research Centers (IRCs). The total number of severe pneumonia cases was 11 (8 Guatemala, 0 Peru, 1 Rwanda, 2 India). The observed incidence of severe pneumonia was 5.6 events/100 child-years. Assuming equal person time between the study arms and a relative risk of 0.67 the estimated control group incidence

was 6.7 events/100 child-years (95%CI 3.1, 10.2). The *a priori* background incidence required to be adequately powered assuming a 33% intervention effect size is 6 events/100 child-years. Therefore, the trial's estimated control group incidence based on blinded data (6.7 events/100 child-years) was likely meeting the target incidence (6 events/100 child-years). Meeting participants observed from the trial data that the surveillance activities at Guatemala were identifying and screening more study children with acute illnesses. Participants further observed that the incidence of severe pneumonia was much higher in Guatemala relative to the other IRCs.

PINS (Pneumonia International Network Surveillance) data:

The stated purpose of the PINS network was to understand pneumonia incidence and severity at the four IRCs given this was unknown prior to the trial. The surveillance system was designed to help determine, in parallel to the main trial, whether the number of severe pneumonia cases in the IRC study areas would likely be sufficient or if additional recruitment or other adaptions will be necessary. All IRCs other than Guatemala completed the surveillance data collection retrospectively through medical chart extraction. Guatemala collected data prospectively, mainly through HAPIN study staff. All IRCs reported surveillance data from a period of at least 17 months, with Rwanda reporting data from 30 months. All the IRC PINS incidence estimates were lower than the 6 events/100 child-years background target rate, except for Guatemala. Furthermore, all the IRC PINS incidence estimates were lower than the observed HAPIN trial data incidence. Consistent with the HAPIN trial data incidence, the Guatemala PINS incidence estimates were higher than the other IRCs. Lastly, the

oxygen saturation variable was recorded in most medical charts, and it was concluded that extraction of this variable from the medical chart could be considered for inclusion in the trial data since IRCs were reporting challenges in obtaining this measurement when the child was receiving supplemental oxygen.

Analysis of Guatemala IRC:

Additional analyses were conducted to evaluate why Guatemala was observed to have a higher incidence of severe pneumonia and why surveillance activities were identifying higher numbers of acutely ill children. Analyses comparing the case characteristics of pneumonia cases in Guatemala, compared to the other IRCs, showed no substantive differences that clearly indicated possible systematic deviations from the trial protocol when screening and evaluating children for severe pneumonia. It was concluded that the trial surveillance in Guatemala, mainly due to 24 hours a day and 7 days per week staffing, was likely to be more comprehensive than currently administered at the other IRCs. It was also observed that Guatemala's trial incidence is comparable to other published incidence rates from children with pneumonia in Guatemala, and meeting participants concluded that it is likely that the incidence of severe pneumonia in Guatemala is simply higher than in the other IRCs.

Evaluation of potential revised pneumonia definitions using HAPIN data:

We evaluated amended pneumonia definitions since the current pneumonia case definition was inconsistent with the World Health Organization case definition of pneumonia, as well as other landmark child pneumonia studies (e.g., PERCH), and this

may limit external validity and generalizability of the trial findings. Notably, the two main features required of the pneumonia case definition for this trial are severity, to optimize public health impact of the trial by targeting cases at greatest risk of mortality, and objective diagnosis, to prioritize specificity and limit inclusion of non-pneumonia cases. Four amended case definitions were assessed. Compared to the current pneumonia case definition, analyses demonstrated that the WHO pneumonia case definition, when enriched by confirmatory imaging and physiologic oxygen saturation thresholds, increased case severity, provided an incidence of 12.4 events/100 child-years (95%CI 7.8, 17.0) and power of 0.89 assuming a relative risk of 0.67. This amended definition maintained 0.80 power at a relative risk of 0.73. The two key aspects of the case definition were improved using this amended definition, severity and objective diagnosis (through imaging and pulse oximetry).

Other options discussed:

Multiple other options were evaluated and presented to the meeting participants, including no protocol change, no amendment to the case definition but an increase in sample size (across all IRCs and also only in Guatemala), amend the case definition and increase sample size, a combination of the above but with revised surveillance, and amending the pneumonia endpoint to a secondary trial outcome.

Final external expert advisor recommendations: As the current follow-up time was <1% the external expert advisors recommended the following:

1. Amend the case definition to be consistent with the current WHO severe pneumonia definition and conduct confirmatory imaging on non-hypoxemic cases
2. Revise the oxygen saturation threshold to physiologic levels (≤92% at Guatemala, India, Rwanda; ≤86% at Peru)
3. Children can be considered to have severe hypoxemic pneumonia if observed or documented to be receiving mechanical ventilation, high flow nasal cannula oxygen, or non-invasive ventilation with CPAP or BiPAP. The oxygen saturation variable can be extracted from the medical chart and the same thresholds detailed in recommendation #2 applied for defining hypoxemia. Death attributed to pneumonia should be included in the case definition.
4. Strengthen pneumonia surveillance at the Peru, India, and Rwanda IRCs using existing resources.

Verbal autopsy methodology

Purpose:

The Household Air Pollution Intervention Network (HAPIN) Trial aimed to assess the impact of a liquefied petroleum gas (LPG) cooking stove and fuel intervention on health. This was done in four countries: Guatemala, India, Peru and Rwanda. One of the health outcomes that were recorded was deaths amongst children under-five years. The purpose of this exercise is to determine which of these deaths died of probable pneumonia.

Methods:

The verbal autopsy data will be analyzed using three methods, to produce 3 different primary cause of death variables. The primary cause of death will be described for PCVA and CCVA methods, and the three binary variables for “probable pneumonia death” will be described using proportions by country and age group.

1. Physician Coded verbal autopsy (PCVA)

Two physicians, blinded to both study arms, each other’s assessments and any existing cause of death classification were asked to independently review the open narrative and close questions from the VAs. To be eligible, the physicians needed to be actively working in the study country and not have been a member of the HAPIN study team. They were asked to assign at least one cause of death (primary), and if they deem appropriate, could assign a secondary cause. In cases where the primary cause of death was discrepant between the two physicians, a third independent physician review was done. The third reviewer had access to the cause of death allocation and was

asked to arbitrate between these – or they could assign a new and different primary cause of death. In cases where no consensus could be reached, the VA was classified as “99 Undetermined”, in line with previously published PCVA protocols[5]. The codes for cause of death were the ICD-10 World Health Organisation 2016 list (Table S6).
A binary variable was generated with 1=probable pneumonia death and 0=unlikely pneumonia death, if either the primary or secondary cause of death was coded as “10.03 Neonatal pneumonia” or “01.02 Acute respiratory infection, including pneumonia”.

2. Computer coded verbal autopsy (CCVA)

The closed questions from the verbal autopsies were analysed using the WHO’s OpenVA platform[6]. This includes multiple automated coding algorithms, and the cause of death from the InterVA-5 algorithm will be presented. This algorithm is based on Bayesian statistics, which assign a probable fraction for each cause of death, based on pre-specified weights assigned to each sign and symptom reported. The variables from the HAPIN VA tool were mapped to the WHO’s 2016 VA tool.

The algorithms require each country to be classified as high (>1%), low (0.1-1%) or very low (<0.1%) in terms of HIV/AIDs and malaria mortality. The countries were classified based on 2019 Global Burden of Disease data as follows: Guatemala: HIV/AIDS = low, malaria = very low; Peru: HIV/AIDS = high, malaria = very low; India: HIV/AIDS = low, malaria = low; Rwanda: HIV/AIDS = high, malaria = high. The following months were classified as being the rainy season in each country: Guatemala: May-October; Peru:

November-March; India: June-September; Rwanda: March-May. InterVA5 requires both an age and sex to process the data. The neonatal VAs did not contain child sex, and therefore this was imputed as Male for all neonatal deaths; a sensitivity analysis using Female was conducted to check if the distribution of deaths changed.

A binary variable was generated with 1=probable pneumonia death and 0=unlikely pneumonia death, if either the primary or secondary cause of death was coded as "10.03 Neonatal pneumonia" or "01.02 Acute respiratory infection, including pneumonia".

3. Symptom coded

Using the two questions from the closed questions in the WHO VA tool: "Did baby/child have any difficulty with breathing?" and "Did baby/child have cough?", a binary variable will be created for probable pneumonia, using the same approach as WHO's IMCI algorithm. A child with a cough and/or difficult breathing and either fast breathing or chest in-drawing were classified as "1=probable pneumonia death".

<u>Findings:</u>

Overall, there were 61 VAs, with 38 (62%) neonatal deaths and 23 (38%) infant deaths. A summary of the clinician coding process is presented in Table S2, and the primary cause of death assignment from both the algorithm and clinicians in presented in Table S3. For probable pneumonia deaths, using the symptom coding approach resulted in the largest number of deaths defined as "probable pneumonia" (25%), while the InterVA-5 coded approach the fewest (14%) – Table S4.

Comment on agreement:

Overall, the physician agreement observed in this exercise is comparable to published evaluations of agreement in child VA coding. We found that for 35/61 (57%) of VAs, there was agreement in the primary cause of death assigned by two independent physicians – and this was consistent with agreement for the sub-group who were eventually classified as probable pneumonia deaths (58%, 7/12). When a third physician reviewed those without agreement, this increased to 50/61 (82%). We observed variation in agreement by study setting and by death type, with higher disagreement from Guatemala and Peru and in infants versus neonates. The cause with highest agreement was birth asphyxia (91%). Other studies have reported similar rates: 60% agreement in infectious causes[7]; 65% agreement for neonates and 72% for infants[8]; 51% of deaths without a cause assigned due to disagreement[9].

For PCVA and CCVA agreement, this has been found to be poor in deaths amongst children and neonates, and for infectious deaths – with different methods to cause of death assignment performing better for some causes than others[10,11]. Therefore, the lack of agreement in this exercise is not unexpected. Overall, 4/12 (33%) physician coded probable pneumonia deaths were also classified as pneumonia deaths using the InterVA5 algorithm. This is low, but the HAPIN VA tool did not completely map to the WHO 2016 tool which the InterVA-5 Bayesian algorithm is based on, and likely resulted in poorer performance.

Available from: Appendix 3 – Guidance for interpreting VA results (version 0120-01), available from: https://www.who.int/standards/classifications/other-classifications/verbal-autopsy-standards-ascertaining-and-attributing-causes-of-death-tool.

III. Tables

Table S1. Secondary outcome definitions

WHO IMCI pneumonia	WHO IMCI non-severe and severe pneumonia
WHO IMCI severe pneumonia	Cough and/or difficult breathing and<br>• any general danger sign (unable to drink or breastfeed, vomiting everything, convulsions, lethargic or unconscious) or<br>• stridor at rest or<br>• severe acute malnutrition or<br>• HIV infection or exposure (if chest indrawing also) or<br>• Hypoxemia (SpO_2 <90%)
WHO IMCI non-severe	Cough and/or difficult breathing and<br>• any general danger sign (unable to drink or breastfeed, vomiting everything, convulsions, lethargic or unconscious) or<br>• stridor at rest or<br>• severe acute malnutrition or<br>• HIV infection or exposure (if chest indrawing also) or<br>• Hypoxemia (SpO_2 <90%)
WHO Pocketbook pneumonia	Pocketbook non-severe and severe pneumonia
WHO Pocketbook severe pneumonia	Cough and/or difficult breathing and<br>• central cyanosis or SpO_2<90% or<br>• severe respiratory distress (grunting, very severe chest indrawing) or sign of pneumonia (fast breathing, chest indrawing, or lung auscultation signs of decreased or bronchial breath sounds, crackles, abnormal vocal resonance, pleural rub) and at least one general danger sign (unable to drink or breastfeed, vomiting everything, convulsions, lethargic or unconscious) or other high-risk condition (severe acute malnutrition or HIV infection or exposure)
WHO Pocketbook non-severe pneumonia	Cough and/or difficult breathing and<br>• chest indrawing or fast breathing and<br>• no sign of Pocketbook WHO severe pneumonia
Hypoxemia and/or imaging-confirmed pneumonia	Either primary endpoint pneumonia by lung ultrasound or chest radiograph imaging or hypoxemia (categorized as SpO_2 <93% for Guatemala, India, and/or Rwanda, and <87% for Peru)

Hypoxemic pneumonia	Categorized as SpO_2 <93% for Guatemala, India, and/or Rwanda, and <87% for Peru
Imaging-confirmed pneumonia	Primary endpoint pneumonia by either lung ultrasound or chest radiography
Hospitalized respiratory illness	Hospitalization for respiratory illness at any time during the follow-up period

WHO indicates World Health Organization; IMCI, Integrated Management of Childhood Illness; SpO_2, peripheral oxyhemoglobin saturation.

Table S2. Background information on the broader population affected by household air pollution

Category	Information
**Disease, problem, or condition under investigation**	Infant severe pneumonia
**Special considerations related to:**	
**Sex and gender**	Infant severe pneumonia affects girls and boys similarly.[12]
**Age**	Incidence of severe pneumonia is much higher during the first year of life than during later childhood or adulthood.[12]
**Race or ethnic group**	Latino, Black and Asian persons may be disproportionately more affected by severe pneumonia worldwide when compared to whites.
**Geography**	Infants in resource-poor settings of Africa, Asia and Latin America are disproportionally more affected by severe pneumonia than children in Europe, North America and Australasia. Areas of Latin America, Africa and Southeast Asia are the most heavily impacted by exposure to household air pollution.[13] LRI incidence is higher in Guatemala than most other countries (173 per thousand per year) and is higher in Rwanda (113 per thousand per year) than it is in India (54 per thousand per year) and Peru (97 per thousand per year). Guatemala, Rwanda, and India have LRI death rates (3.8-4.8 per 1000 per year) similar to low-middle-income countries on average, whereas Peru has lower LRI incidence (1.4 per 100 per year) that is similar to the middle-income countries on average.
**Other considerations**	Household air pollution is estimated to cause approximately 423,000 LRI deaths per year, and 152,000 (36%) of these deaths occur during the first year of life.[12] Within regions and countries, populations most impacted by HAP often include indigenous populations and lower-income populations.[13]
**Overall representativeness of this trial**	We studied the infant age range, which has the greatest burden of severe pneumonia attributable to HAP. Pregnant women were enrolled from obstetric clinics or national registries of pregnant women, which are representative of the population of pregnant women in the rural settings under study. The relative incidence rates of severe pneumonia in this trial by site were generally consistent the relative incidence and mortality rates of LRI from the global burden of disease study.[12]

Table S3. Air pollution exposure measurements

Characteristic		Intervention (N=1536)	Control (N=1525)
Baseline $PM_{2.5}$ ($\mu g/m^3$)[1]	Mean (SD)	119.5 (133.6)	111.9 (110.9)
	Missing, n	184	173
	N	1352	1352
Baseline Black carbon ($\mu g/m^3$)[1]	Mean (SD)	12.6 (10.9)	12.4 (9.2)
	Missing, n	313	314
	N	1223	1211
Baseline Carbon monoxide (ppm)[1]	Mean (SD)	2.7 (4.5)	2.3 (4.0)
	Missing, n	152	150
	N	1384	1375
Trial-period antenatal $PM_{2.5}$ ($\mu g/m^3$)[2]	Mean (SD)	35.0 (37.2)	103.3 (97.9)
	Missing, n	99	116
	N	1437	1409
Trial-period postnatal $PM_{2.5}$ ($\mu g/m^3$)[2]	Mean (SD)	37.9 (97.4)	109.2 (122.7)
	Missing, n	688	592
	N	848	933
Trial-period antenatal black carbon ($\mu g/m^3$)[2]	Mean (SD)	4.1 (5.6)	11.2 (9.3)
	Missing, n	123	149
	N	1413	1376
	Mean (SD)	0.7 (1.2)	2.2 (3.6)
	Missing, n	86	95

Trial-period antenatal carbon monoxide (ppm)[2]	N	1450	1430
Trial-period postnatal carbon monoxide (ppm)[2]	Mean (SD)	0.9 (2.0)	2.6 (4.1)
	Missing, n	571	609
	N	965	916

IRC indicates International Research Center; SD, standard deviation; PM, particulate matter; IQR, interquartile range.

[1]Missing includes invalid samples that failed to pass quantitative quality checks, including samples with unacceptable flow rates, filter damage, and measurement durations outside of 24 ± 4 hours.

[2]Trial-period measurements refer to post-randomization pollutant values, which are presented as the median of the average of household-level measures. Missing includes invalid samples that failed to pass quantitative quality checks, including samples with unacceptable flow rates, filter damage, and measurement durations outside of 24 ± 4 hours.

Table S4. Verbal Autopsy – Summary of physician coding process

	India	Rwanda	Guatemala	Peru
Total VAs	12 deaths	16 deaths	20 deaths	13 deaths
PCVA agreement	8/12 (67%)	13 /16 (81%)	9/20 (45%)	5/13 (38%)
Cause assigned	10/12 (83%)	15 /16 (94%)	17/20 (85%)	8/13 (62%)

Table S5. Verbal Autopsy – Primary cause of death, assigned from different methods

ICD-10 cause of death category		PCVA	CCVA
Neonatal (n=38)	10.01 Prematurity or low birth weight	8 (21%)	15 (40%)
	10.02 Birth asphyxia (includes hypoxia and respiratory distress)	11 (29%)	10 (26%)
	10.03 Neonatal pneumonia	4 (11%)	2 (5%)
	10.04 Neonatal sepsis	6 (16%)	6 (16%)
	10.06 Congenital malformation	1 (3%)	3 (8%)
	10.99 Other and unspecified perinatal cause	1 (3%)	-
	11.01 Fresh stillbirth	-	1 (3%)
	98 Other and unspecified NCD	1 (3%)	-
	99 Undetermined	6 (16%)	1 (3%)
Infant (n=23)	01.01 Sepsis (non-obstetric)	3 (13%)	1 (4%)
	01.02 Acute respiratory infection, including pneumonia	6 (26%)	5 (22%)
	01.04 Diarrhoeal diseases	3 (13%)	8 (35%)
	01.05 Malaria	-	1 (4%)
	01.07 Meningitis and encephalitis	1 (4%)	-
	1.99 Other and unspecified infectious disease	-	1 (4%)
	03.02 Severe malnutrition	-	1 (4%)
	04.01 Acute cardiac disease	1 (4%)	-
	10.06 Congenital malformation	2 (9%)	-
	99 Undetermined	6 (26%)	4 (17%)

Table S6. Verbal Autopsy – Probable pneumonia deaths, assigned through different verbal autopsy analysis methods

		PCVA<br>n (%)	CCVA<br>n (%)	Symptom coded<br>n (%)
Total (n=61)		12 (20%)	8 (14%)	15 (25%)
Study country	Peru	6 (46%)	2 (17%)	5 (38%)
	Guatemala	4 (20%)	2 (11%)	6 (30%)
	Rwanda	0 (0%)	1 (6%)	3 (19%)
	India	2 (17%)	3 (25%)	1 (8%)
Age group	<28 days	6 (16%)	2 (5%)	9 (24%)
	1-11 months	6 (26%)	6 (26%)	6 (26%)

PCVA: Physician coded VA; CCVA: computer coded VA using InterVA5

Table S7. Verbal Autopsy – Output data dictionary

A single .csv file contains all the cause of death codes (HAPIN_CoD_alldata.csv).

Variable name	Definition	Format/Coding
irc	Study country	String
hhid_blinded	Unique study ID	String
symptomCoD	Probable pneumonia death, defined using the IMCI approach from the VA closed questions	0 = unlikely pneumonia;<br>1 = probable pneumonia
cough	WHO VA question: Did baby/child have cough?	Yes; No; DK
breath	WHO VA question: Did baby/child have any difficulty with breathing?	Yes; No; DK
breath_fast	WHO VA question: Did baby/child have fast breathing?	Yes; No; DK
indraw	WHO VA question: Did baby/child have in-drawing of the chest?	Yes; No; DK
pcvaCoD	Probable pneumonia death, based on primary or secondary cause assigned through agreed physician review	0 = unlikely pneumonia;<br>1 = probable pneumonia
PrimaryCOD_FINAL	ICD-10 coded final agreed primary cause of death from physician review	String
SecondaryCOD_FINAL	ICD-10 coded final agreed secondary cause of death from physician review	String
PrimaryCOD_1	ICD-10 coded primary cause of death from first physician review	String
SecondaryCOD_1	ICD-10 coded secondary cause of death from first physician review	String
PrimaryCOD_2	ICD-10 coded primary cause of death from second physician review	String
SecondaryCOD_2	ICD-10 coded secondary cause of death from second physician review	String
PrimaryCOD_3	ICD-10 coded primary cause of death from third physician review (only cases where there was no agreement between)	String
SecondaryCOD_3	ICD-10 coded secondary cause of death from third physician review (only cases where there was no agreement between)	String
agreement		0=no<br>1=yes

ccvaCoD	Probable pneumonia death, based on primary or secondary cause assigned through InterVA-5 automated analysis	0 = unlikely pneumonia;<br>1 = probable pneumonia
ccva_cause1	Primary cause of death assigned by InterVA-5	String
ccva_lik1	Likelihood of cause classification	Decimal
ccva_cause2	Secondary cause of death assigned by InterVA-5	String
ccva_lik2	Likelihood of cause classification	Decimal

Table S8. Verbal Autopsy – ICD-10 derived cause of death codes

Neonatal Deaths	
10.01	Prematurity or low birth weight
10.02	Birth asphyxia (includes hypoxia and respiratory distress)
10.03	Neonatal pneumonia
10.04	Neonatal sepsis
10.05	Neonatal tetanus
10.06	Congenital malformation
10.99	Other and unspecified perinatal cause
99	Undetermined
Post-Neonatal Deaths	
01.01	Sepsis (non-obstetric)
01.02	Acute respiratory infection, including pneumonia
01.03	HIV/AIDS related death
01.04	Diarrhoeal diseases
01.05	Malaria
01.06	Measles
01.07	Meningitis and encephalitis
01.08	Tetanus
01.09	Pulmonary tuberculosis
01.10	Pertussis
01.11	Haemorrhagic fever
01.99	Other and unspecified infectious disease
02	Neoplasm (specify type in notes)
03.01	Severe anaemia
03.02	Severe malnutrition
03.03	Diabetes mellitus
04.01	Acute cardiac disease
04.03	Sickle cell with crisis
04.02	Stroke
04.99	Other and unspecified cardiac disease
05.01	Chronic obstructive pulmonary disease
05.02	Asthma
06.01	Acute abdomen
06.02	Liver cirrhosis
07.01	Renal failure
08.01	Epilepsy
98	Other and unspecified NCD
12	Accident and trauma (specify type in notes)
99	Undetermined

Table S9. Characteristics of severe pneumonia cases (primary outcome) by study group

Characteristic		Intervention (N=85)	Control (N=90)
Demographic characteristics			
IRC, n (%)	Guatemala	38 (44.7%)	32 (35.6%)
	India	5 (5.9%)	6 (6.7%)
	Peru	7 (8.2%)	6 (6.7%)
	Rwanda	35 (41.2%)	46 (51.1%)
Age (months)	Median (IQR)	2.1 (0.8, 7.6)	2.8 (0.9, 7.8)
	<2	39 (45.9%)	37 (41.1%)
	2 to <6	17 (20.0%)	25 (27.8%)
	6 to <12	29 (34.1%)	28 (31.1%)
Sex, n (%)	Male	47 (55.3%)	41 (45.6%)
	Female	38 (44.7%)	49 (54.4%)
Vaccination characteristics			
Up-to-date pentavalent vaccine status at diagnosis[1]	n (%)	76 (90.5%)	75 (94.9%)
	Missing, n (%)	1 (1.2%)	11 (12.2%)
Up-to-date pneumococcal conjugate vaccine status at diagnosis[1]	n/N (%)	72 (91.1%)	70 (93.3%)
	Missing, n (%)	1 (1.2%)	9 (10.7%)
Exposure characteristics			
Baseline $PM_{2.5}$ (μg/m^3)	Median (IQR)	120.9 (67.6, 201.0)	98.9 (54.2, 161.9)
	Invalid, n (%)	5 (5.9%)	6 (6.7%)
	Missing, n (%)	2 (2.4%)	4 (4.4%)
Antenatal $PM_{2.5}$ (μg/m^3)	Median (IQR)	29.5 (22.3, 48.1)	92.5 (52.5, 134.5)
	Invalid, n (%)	1 (1.2%)	1 (1.1%)
	Missing, n (%)	0	2 (2.2%)
Postnatal $PM_{2.5}$ (μg/m^3)	Median (IQR)	24.5 (18.7, 42.3)	91.0 (41.9, 199.7)
	Missing, n (%)	34 (40.0%)	32 (35.6%)
Baseline black carbon (μg/m^3)	Median (IQR)	13.0 (10.1, 17.3)	12.6 (7.8, 16.1)
	Invalid, n (%)	19 (22.4%)	18 (20.0%)
	Missing, n (%)	2 (2.4%)	4 (4.4%)
Antenatal black carbon (μg/m^3)	Median (IQR)	3.7 (2.7, 5.1)	11.7 (7.5, 15.4)

	Invalid, n (%)	3 (3.5%)	3 (3.3%)
	Missing, n (%)	0	2 (2.2%)
Baseline carbon monoxide (ppm)	Median (IQR)	1.4 (0.7, 3.1)	1.0 (0.5, 1.9)
	Invalid, n (%)	7 (8.2%)	8 (8.9%)
	Missing, n (%)	3 (3.5%)	2 (2.2%)
Antenatal carbon monoxide (ppm)	Median (IQR)	0.4 (0.1, 0.8)	1.2 (0.7, 2.3)
	Invalid, n (%)	0	0
	Missing, n (%)	0	4 (4.4%)
Postnatal carbon monoxide (ppm)	Median (IQR)	0.4 (0.1, 0.8)	1.9 (0.8, 3.5)
	Missing, n (%)	31 (36.5%)	33 (36.7%)
Clinical characteristics			
Weight (kg)	Median (IQR)	5.6 (3.6, 7.6)	5.6 (3.5, 7.6)
	Missing, n (%)	6 (7.0%)	9 (10.0%)
Severe acute malnutrition[2]	n (%)	9 (11.7%)	7 (9.1%)
	Missing, n (%)	8 (9.4%)	13 (14.4%)
Temperature >38° C	n (%)	30 (53.6%)	45 (66.2%)
	Missing, n (%)	30 (37.0%)	45 (52.9%)
Respiratory rate, breaths/min	Median (IQR)	59.5 (49.0, 65.5)	60.5 (46.0, 67.2)
	Missing, n (%)	5 (5.9%)	6 (6.7%)
SpO_2[3]	Median (IQR)	89.2% (84.2, 91.3)	89.3% (86.0, 91.3)
	Missing, n (%)	13 (15.3%)	17 (18.9%)
Hypoxemia[4]	n (%)	69 (85.2%)	72 (84.7%)
	Missing, n (%)	4 (4.7%)	5 (5.5%)
Wheeze +/- crackles on chest auscultation	n (%)	17 (21.8%)	17 (20.7%)
	Missing, n (%)	7 (8.2%)	8 (8.9%)
At least one respiratory danger sign[5]	n (%)	58 (76.3%)	64 (80.0%)
	Missing, n (%)	4 (4.7%)	10 (11.1%)
Chest indrawing	n (%)	39 (48.8%)	42 (50.0%)
	Missing, n (%)	5 (5.9%)	6 (6.7%)
Head nodding	n (%)	18 (22.5%)	25 (29.8%)

	Missing, n (%)	5 (5.9%)	6 (6.7%)
Persistent nasal flaring	n (%)	12 (15.0%)	21 (25.0%)
	Missing, n (%)	5 (5.9%)	6 (6.7%)
Grunting	n (%)	12 (18.2%)	5 (6.9%)
	Missing, n (%)	19 (22.3%)	18 (20%)
Stridor when calm	n (%)	12 (15.0%)	7 (7.8%)
	Missing, n (%)	5 (5.9%)	6 (6.7%)
Audible wheeze	n (%)	13 (16.7%)	13 (15.9%)
	Missing, n (%)	7 (8.2%)	8 (8.9%)
Tracheal tugging	n (%)	22 (27.5%)	39 (46.4%)
	Missing, n (%)	5 (5.9%)	6 (6.7%)
Intercostal retractions	n (%)	20 (25.0%)	22 (26.2%)
	Missing, n (%)	5 (5.9%)	6 (6.7%)
At least one general danger sign[6]	n (%)	34 (44.7%)	32 (38.6%)
	Missing, n (%)	9 (10.6%)	6 (6.7%)
Unable to drink or breastfeed	n (%)	10 (13.2%)	14 (17.1%)
	Missing, n (%)	9 (10.6%)	8 (8.9%)
Vomiting everything	n (%)	7 (9.0%)	8 (9.8%)
	Missing, n (%)	7 (8.2%)	8 (8.9%)
Convulsions	n (%)	5 (6.2%)	3 (3.6%)
	Missing, n (%)	5 (5.9%)	6 (6.7%)
Lethargy or unconscious	n (%)	13 (16.2%)	12 (14.3%)
	Missing, n (%)	5 (5.9%)	6 (6.7%)
At least one neonatal danger sign[7]	n (%)	22/30 (73.3%)	20/35 (57.1%)
	Missing, n (%)	9 (23.1%)	2 (5.4%)
Unable to feed well	n (%)	13 (48.1%)	8 (24.2%)
	Missing, n (%)	12 (30.8%)	4 (10.8%)
Not moving at all or moves only	n (%)	5 (14.7%)	4 (11.4%)
	Missing, n (%)	3 (7.6%)	2 (5.4%)

Grunting	n (%)	6 (17.6%)	4 (11.4%)
	Missing, n (%)	3 (7.6%)	2 (5.4%)
Severe chest indrawing	n (%)	5 (14.7%)	11 (31.4%)
	Missing, n (%)	3 (7.6%)	2 (5.4%)
Imaging positive[8]	n (%)	43 (57.3%)	47 (60.2%)
	Missing, n (%)	10 (11.8%)	12 (13.3%)
Hospitalized	n (%)	47 (58.0%)	43 (51.4%)
	Missing, n (%)	4 (4.7%)	6 (6.7%)
Oxygen treatment	n (%)	26 (32.1%)	28 (32.9%)
	Missing, n (%)	4 (4.7%)	5 (5.5%)
Advanced respiratory supportive care[9]	n (%)	8 (9.9%)	15 (17.6%)
	Missing, n (%)	4 (4.7%)	5 (5.5%)
Mortality	n (%)	4 (4.7%)	8 (8.8%)

IRC indicates International Research Center; IQR, interquartile range; PM, particulate matter; SpO_2, peripheral arterial oxyhemoglobin saturation.

[1]Vaccination status two weeks before achieving case status. Pentavalent vaccine used was DTwP-HepB+Hib (liquid). Pneumococcal conjugate vaccine was unavailable in India; denominator for up-to-date pneumococcal conjugate vaccine status 84 for intervention and 84 for control participants.

[2]Weight-for-length z score <-3 or if a length measurement is unavailable then weight-for-age z score <-3. For <60-day olds either weight-for-length z score <-3 or weight-for-age z score <-3 used.

[3]Average of measurements when multiple measurements available.

[4]SpO_2<93% for Guatemala, India, or Rwanda; SpO_2<87% for Peru; or if required advanced respiratory care – non-invasive or invasive ventilation.

[5]Any of the following: head nodding, persistent nasal flaring, grunting, stridor when calm, audible wheeze, tracheal tugging, chest indrawing, severe chest indrawing, or intercostal recessions.

[6]Any of the following: unable to drink or breastfeed, vomiting everything, convulsions, lethargy or unconscious, stridor while calm.

[7]Any of the following if <2 months of age: unable to feed, not moving at all or moves with stimulation only, grunting, or severe chest indrawing.

[8]Intervention arm: 42/61 (68.9%) and 1/14 (7.1%) had positive lung ultrasound and chest radiograph imaging. Control arm: 44/68 (64.7%) and 3/10 (30.0%) had positive ultrasound and chest radiograph imaging.

[9]Any of the following: non-invasive ventilation (high flow oxygen, continuous positive airway pressure, bi-level positive airway pressure), or invasive mechanical ventilation.

Table S10. Time to event analysis – secondary analysis

		Intervention	Control
HAPIN pneumonia: Only the first pneumonia event	Events	80	80
	Number of children	1525	1536
	Hazard ratio (95% CI)	1.01 (0.74, 1.38)	Reference
HAPIN pneumonia: All the pneumonia events	Events	85	90
	Number of children	1525	1536
	Hazard ratio (95% CI)	0.96 (0.70, 1.32)	Reference
All-cause mortality	Events	28	36
	Number of children	1525	1536
	Hazard ratio (95% CI)	0.77 (0.47, 1.27)	Reference
All-cause mortality in >30 days	Events	9	16
	Number of children	1512	1502
	Hazard ratio (95% CI)	0.56 (0.25, 1.27)	Reference
All-cause mortality in <30 days	Events	19	20
	Number of children	1536	1525
	Hazard ratio (95% CI)	0.95 (0.51, 1.77)	Reference
Pneumonia deaths assessed by verbal autopsy	Events	4	8
	Number of children	1536	1525
	Hazard ratio (95% CI)	0.48 (0.15, 1.61)	Reference

Table S11. Primary pneumonia analysis accounting for COVID-19 period and child´s age – secondary analysis

Analysis	Variable	Exponentiated coefficients (95% CI)	
		All IRCs[1]	Only Rwanda
Unadjusted	Intervention	0.96 (0.70, 1.32)	0.80 (0.50, 1.29)
Adjusted for COVID period	Intervention	0.96 (0.70, 1.32)	0.81 (0.50, 1.3)
	Pre-COVID	4.33 (2.58, 7.27)	1.86 (0.99, 3.51)
Interaction with COVID period	Intervention – Pre-COVID	1.08 (0.77, 1.51)	0.94 (0.55, 1.60)
	Intervention – During-COVID	0.47 (0.19, 1.19)	0.48 (0.16, 1.41)
	Pre-COVID	3.08 (1.56, 6.06)	1.43 (0.62, 3.30)
	Interaction term	2.27 (0.85, 6.10)	1.97 (0.58, 6.70)
Adjusted for COVID period and age.<br><br>(Categorical age)	Intervention	0.96 (0.70, 1.31)	0.81 (0.50, 1.3)
	Pre-COVID	3.82 (2.20, 6.61)	2.27 (1.13, 4.55)
	0m-1m	Reference	Reference
	2m-3m	0.26 (0.16, 0.43)	0.26 (0.11, 0.62)
	4m-5m	0.42 (0.27, 0.65)	0.37 (0.17, 0.83)
	6m-7m	0.21 (0.11, 0.40)	0.40 (0.18, 0.89)
	8m-9m	0.37 (0.22, 0.64)	0.72 (0.35, 1.49)
	10m-11m	0.73 (0.45, 1.18)	1.47 (0.77, 2.83)
Adjusted for COVID period and age.<br><br>(Numeric age modeled with splines: ns(AgeAtStart_d,5))	Intervention	0.96 (0.70, 1.31)	0.81 (0.50, 1.30)
	Pre-COVID	3.68 (2.08, 6.49)	2.21 (1.08, 4.53)
	AgeAtStart_d, 1	0.24 (0.10, 0.56)	0.15 (0.04, 0.60)
	AgeAtStart_d, 2	0.10 (0.04, 0.28)	0.45 (0.12, 1.68)
	AgeAtStart_d, 3	0.38 (0.16, 0.94)	0.55 (0.18, 1.70)
	AgeAtStart_d, 4	0.04 (0.01, 0.14)	0.26 (0.04, 1.68)
	AgeAtStart_d, 5	1.95 (0.89, 4.26)	3.33 (1.29, 8.62)
Interaction with COVID period and adjusted for age	Intervention – Pre-COVID	1.07 (0.77, 1.50)	0.94 (0.55, 1.59)
	Intervention – During-COVID	0.47 (0.19, 1.19)	0.48 (0.16, 1.42)
	Pre-COVID	2.72 (1.35, 5.46)	1.75 (0.73, 4.18)
	0m-1m	Reference	Reference
	2m-3m	0.26 (0.16, 0.43)	0.26 (0.11, 0.62)
	4m-5m	0.42 (0.27, 0.65)	0.37 (0.17, 0.84)
	6m-7m	0.21 (0.11, 0.40)	0.40 (0.18, 0.89)
	8m-9m	0.37 (0.22, 0.64)	0.72 (0.35, 1.49)
	10m-11m	0.73 (0.45, 1.18)	1.47 (0.77, 2.82)
	Interaction term	2.27 (0.85, 6.07)	1.95 (0.57, 6.59)

Table S12. Rwanda – secondary analysis

Rwanda period		Intervention	Control
Pre-surveillance in Rwanda (< 2019-Nov-15)	Pneumonia cases	2	3
	Child-years of follow-up	131	140
	Incidence (95% CI) (per 100 child-years)	1.53 (0.38, 6.11)	2.14 (0.70, 6.57)
	Incidence rate ratio (95% CI)	0.71 (0.12, 4.23)	Reference
Post-surveillance in Rwanda (≥ 2019-Nov-15)	Pneumonia cases	33	43
	Child-years of follow-up	234	245
	Incidence (95% CI) (per 100 child-years)	14.1 (9.83, 20.23)	17.55 (12.57, 24.47)
	Incidence rate ratio (95% CI)	0.80 (0.49, 1.31)	Reference

Table S13. Adverse events

<table>
<tr><th rowspan="2">Population</th><th rowspan="2">Relationship of burn to the intervention</th><th rowspan="2">Burn severity</th><th colspan="2">Intervention</th><th colspan="2">Control</th></tr>
<tr><th>Stratified</th><th>Total</th><th>Stratified</th><th>Total</th></tr>
<tr><td rowspan="7">Children</td><td rowspan="2">1 = Definitely related (closely related)</td><td>Non-severe</td><td>0</td><td rowspan="7">3</td><td>0</td><td rowspan="7">7</td></tr>
<tr><td>Severe</td><td>0</td><td>0</td></tr>
<tr><td rowspan="2">2 = Possibly related (maybe related)</td><td>Non-severe</td><td>0</td><td>0</td></tr>
<tr><td>Severe</td><td>0</td><td>0</td></tr>
<tr><td rowspan="2">3 = Not related (clearly NOT related)</td><td>Non-severe</td><td>3</td><td>7</td></tr>
<tr><td>Severe</td><td>0</td><td>0</td></tr>
<tr><td>NA</td><td>NA</td><td>0</td><td>0</td></tr>
</table>

IV. Figures

Figure S1. Primary pneumonia case ascertainment schema

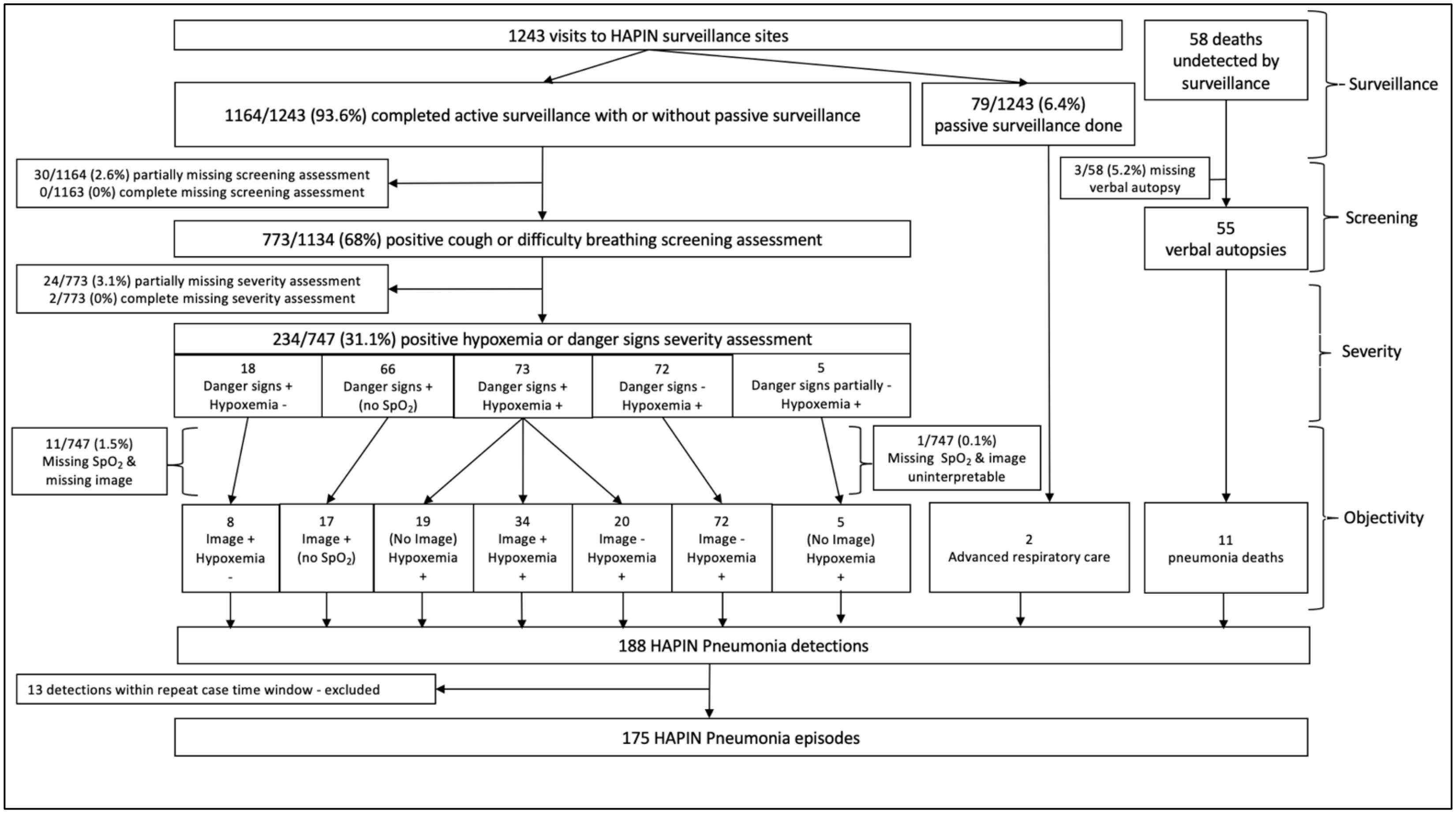

Figure S2. Subgroup analyses of severe pneumonia (primary outcome)

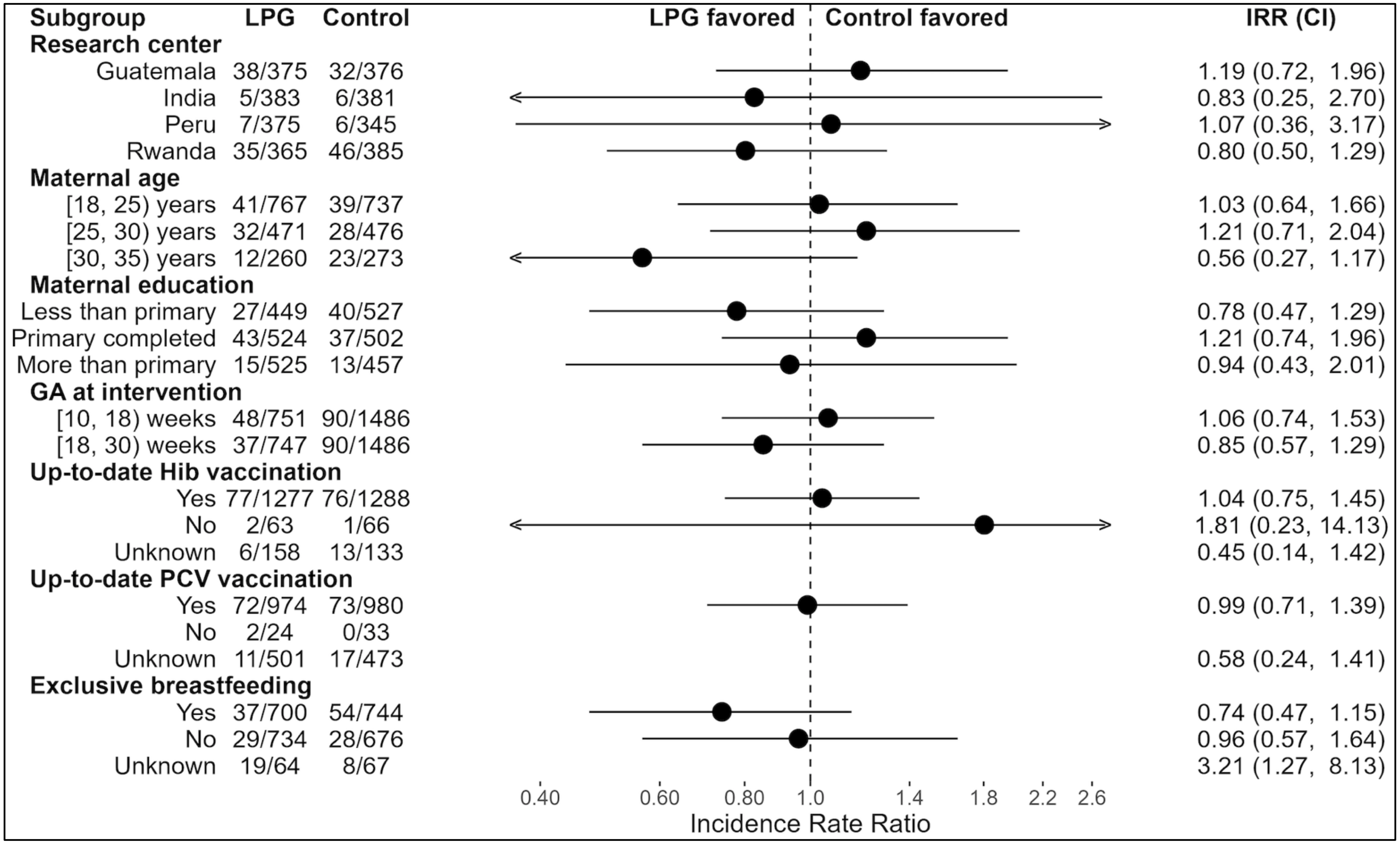

LPG indicates liquefied petroleum gas; IRR, incidence rate ratio; CI, confidence interval; GA, gestational age; Hib, Haemophilus influenzae type b; PCV, pneumococcal conjugate vaccine. The HiB vaccination, PCV vaccination, and exclusive breastfeeding subgroups were determined post-baseline. 95% confidence intervals were not adjusted for multiplicity and should not be used to infer definitive treatment effects.

Figure S3. Episodes and incidence of severe pneumonia (Panel A) and health care visits (Panel B) by COVID-19 period

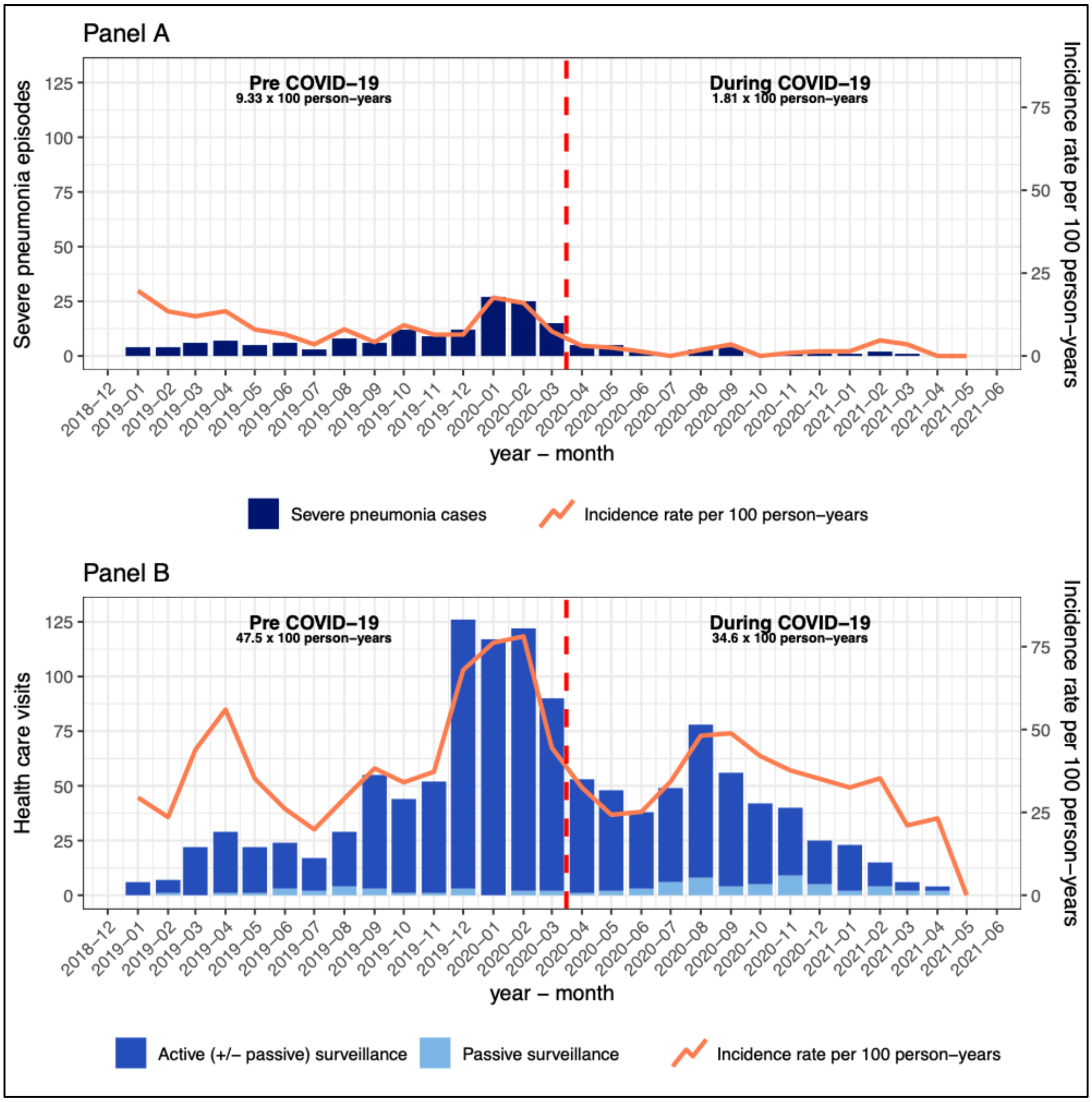

IV. References

1. Clasen T, Checkley W, Peel JL, et al. Design and Rationale of the HAPIN Study: A Multicountry Randomized Controlled Trial to Assess the Effect of Liquefied Petroleum Gas Stove and Continuous Fuel Distribution. *Environ Health Perspect* 2020;128:47008.
2. Johnson MA, Steenland K, Piedrahita R, et al. Air Pollutant Exposure and Stove Use Assessment Methods for the Household Air Pollution Intervention Network (HAPIN) Trial. *Environ Health Perspect* 2020;128:47009.
3. Johnson M, Pillarisetti A, Piedrahita R, et al. Exposure Contrasts of Pregnant Women during the Household Air Pollution Intervention Network Randomized Controlled Trial. *Environ Health Perspect* 2022;130:97005.
4. Liao J, McCracken JP, Piedrahita R, et al. The use of bluetooth low energy Beacon systems to estimate indirect personal exposure to household air pollution. *J Expo Sci Environ Epidemiol* 2020;30:990-1000.
5. Byass P, Kahn K, Fottrell E, Collinson MA, Tollman SM. Moving from data on deaths to public health policy in Agincourt, South Africa: approaches to analysing and understanding verbal autopsy findings. *PLoS Med* 2010;7:e1000325.
6. OpenVA. (https://openva.net/)
7. Aleksandrowicz L, Malhotra V, Dikshit R, et al. Performance criteria for verbal autopsy-based systems to estimate national causes of death: development and application to the Indian Million Death Study. *BMC Med* 2014;12:21.
8. Morris SK, Bassani DG, Kumar R, Awasthi S, Paul VK, Jha P. Factors associated with physician agreement on verbal autopsy of over 27000 childhood deaths in India. *PLoS ONE* 2010;5:e9583.

9. Yé M, Diboulo E, Niamba L, et al. An improved method for physician-certified verbal autopsy reduces the rate of discrepancy: experiences in the Nouna Health and Demographic Surveillance Site (NHDSS), Burkina Faso. *Popul Health Metr* 2011;9:34.

10. Murray CJ, Lozano R, Flaxman AD, et al. Using verbal autopsy to measure causes of death: the comparative performance of existing methods. *BMC Med* 2014;12:5.

11. Leitao J, Desai N, Aleksandrowicz L, et al. Comparison of physician-certified verbal autopsy with computer-coded verbal autopsy for cause of death assignment in hospitalized patients in low- and middle-income countries: systematic review. *BMC Med* 2014;12:22.

12. Stoner, O., Lewis, J., Martínez, I.L. et al. Household cooking fuel estimates at global and country level for 1990 to 2030. *Nat Commun* 2021;12:5793.

13. Institute for Health Metrics and Evaluation. GDD Compare. (http://ihmeuw.org/657c)
